# Preoperative cardiac POCUS in high-risk non-cardiac surgery: A prospective study on anesthetic planning and outcomes

**DOI:** 10.6026/973206300213784

**Published:** 2025-10-31

**Authors:** Swati Deswal, Shantanu Yadav, Ankur jakhar, Mohit Sheoran, Prachi Chauhan, Juhi Mishra

**Affiliations:** 1 Department of Anaesthesia, Varun Arjun Medical College, Shahjahanpur, Uttar Pradesh, India; 2 Department of Orthopedics, SGT Medical College And Research Institute, Gurugram, Haryana, India

**Keywords:** Point-of-care ultrasound, focused transthoracic echocardiography, anesthetic management, high-risk surgery, perioperative outcomes

## Abstract

The role of preoperative cardiac point-of-care ultrasound (POCUS) in modifying anesthetic management and influencing perioperative outcomes in high-risk non-cardiac surgery patients is of interest. A total of 120 adults with Revised Cardiac Risk Index ≥2 or significant cardiac comorbidity underwent standardized preoperative focused transthoracic echocardiography. Clinically significant previously undiagnosed cardiac findings were detected in 31.7% of patients, leading to anesthesia plan modifications in 24.2%, including escalation to invasive monitoring, anesthesia technique changes and preoperative cardiology consultation. Patients whose plans were modified experienced fewer intraoperative hypotensive episodes and reduced unplanned intensive care admissions compared with those without plan changes. Thus, we show the integration of preoperative cardiac POCUS into perioperative assessment pathways for high-risk surgical patients to optimize management and improve hemodynamic stability.

## Background:

Point-of-care ultrasound (POCUS) allows rapid bedside imaging, enabling anesthesiologists to detect clinically significant cardiac abnormalities in the perioperative period [1]. In emergency non-cardiac surgery, a multicenter randomized controlled trial demonstrated that brief, preoperative ultrasound-guided optimization significantly influenced intraoperative management decisions and was associated with improved early outcomes, including reduced 30-day mortality [1]. In the elective high-risk surgical population, the PREOP-FOCUS trial compared preoperative focused cardiac ultrasound (FoCUS) with standard preoperative care and, while no statistically significant difference was observed in composite patient-related outcomes, the trial provided important evidence for selecting outcome measures and estimating sample sizes in future studies [2]. Prospective data from preoperative assessment clinics indicate that anesthesiologist-performed FoCUS frequently alters both diagnosis and anesthetic management. Canty *et al.* reported that in patients with suspected cardiac disease, FoCUS modified the diagnosis in 54% of cases and influenced management in 42% [3]. In the emergency surgical setting, similar work from the same group found changes in diagnosis in 67% of patients and changes in management in 44% after FoCUS [4]. These studies highlight the capacity of FoCUS to uncover previously undetected pathology that may critically influence anesthetic strategy. Early feasibility studies have confirmed that anesthesiologists can reliably perform FoCUS in the perioperative environment. Cowie demonstrated that focused transthoracic echocardiography (TTE) is technically achievable in most patients, with findings that prompted changes in perioperative management in nearly half of the cases studied [5]. Furthermore, the same group later showed that the detection of cardiac pathology through anesthesiologist-performed FoCUS could predict postoperative complications, suggesting its role as a risk-stratification tool [6]. In larger case series, perioperative "rescue echocardiography" performed in hemodynamically unstable patients resulted in a change of management in 59% of cases, including adjustments to fluid therapy, vasoactive medication use and surgical timing [7]. Kratz *et al.* further reported that intraoperative FoCUS in unstable high-risk surgical patients provided new diagnostic information in a significant proportion, leading to targeted hemodynamic interventions [8]. More recently, Wong *et al.* demonstrated that combining cardiac and lung POCUS as part of routine preoperative screening led to anesthesia plan modification in two-thirds of patients, supporting its feasibility and value in broader perioperative risk assessment [9]. Collectively, these studies underscore the diagnostic and management impact of preoperative cardiac POCUS in both elective and emergency surgical settings. They also point to the need for structured integration of POCUS into preoperative workflows, particularly for high-risk patients. Current literature increasingly supports its role in optimizing hemodynamics, guiding anesthetic technique selection and anticipating postoperative complications [10]. Therefore, it is of interest to describe the role of preoperative cardiac POCUS in modifying anesthetic management and its potential impact on perioperative outcomes in high-risk non-cardiac surgery patients.

## Methods:

This was a prospective observational study conducted over a 12-month period in the perioperative setting of high-risk non-cardiac surgeries. Adult patients (≥18 years) scheduled for elective or emergency procedures with a Revised Cardiac Risk Index (RCRI) score ≥2 or documented cardiac comorbidities were eligible. Patients with hemodynamic instability precluding imaging, poor transthoracic echocardiographic windows, or those declining consent were excluded. Preoperative point-of-care cardiac ultrasound was performed by anesthesia consultants trained in focused transthoracic echocardiography. Standardized cardiac views, including parasternal long-axis, parasternal short-axis, apical four-chamber and subcostal views, were acquired using a portable ultrasound machine. Each examination assessed left and right ventricular systolic function, valvular morphology, pericardial effusion and inferior vena cava dimensions for volume status estimation. Findings were documented immediately and communicated to the primary anesthesia team. Any resulting modification in the planned anesthetic technique, monitoring strategy, or perioperative optimization was recorded. All intraoperative hemodynamic events, unplanned intensive care unit admissions and postoperative cardiac complications within 30 days were monitored. Data were collected using a predesigned proforma and analyzed using descriptive and inferential statistics. Continuous variables were expressed as mean ± standard deviation or median with interquartile range. Categorical variables were expressed as frequencies and percentages. Appropriate parametric or non-parametric tests were applied to compare groups and a p-value <0.05 was considered statistically significant.

## Results:

A total of 120 patients were enrolled, with a mean age of 64.2 ± 9.8 years; 72 (60%) were male. Common comorbidities included hypertension (66%), ischemic heart disease (32%) and heart failure (12%). Baseline demographic and clinical characteristics are shown in Table 1 (see PDF). Preoperative cardiac POCUS identified previously undiagnosed significant findings in 38 patients (31.7%), including reduced left ventricular systolic function (n = 14), moderate-to-severe valvular lesions (n = 12), pericardial effusion (n = 6) and markedly dilated inferior vena cava with minimal respiratory variation (n = 6). Anesthetic management was modified in 29 patients (24.2%) based on POCUS findings. The most frequent changes were escalation to invasive arterial monitoring (n = 17), addition of intraoperative trans esophageal echocardiography (n = 5), change of anesthesia technique from general to regional (n = 4) and preoperative cardiology consultation delaying surgery for optimization (n = 3). Patients whose plans were modified had fewer episodes of intraoperative hypotension (13.8% vs 28.6%, p = 0.041) and reduced unplanned ICU admissions (6.9% vs 18.6%, p = 0.048) compared with those without plan changes (Figure 1 - see PDF). Thirty-day postoperative major adverse cardiac events occurred in 5 patients (4.2%), all in the non-modified group.

## Discussion:

This prospective study found that preoperative cardiac point-of-care ultrasound (POCUS) identified clinically significant, previously unrecognized cardiac abnormalities in nearly one-third of high-risk non-cardiac surgical patients. These results mirror earlier observational data from Canty *et al.* where focused transthoracic echocardiography (FoCUS) altered diagnosis in over half of patients and influenced perioperative management in over 40% [3, 4]. The diagnostic yield in our cohort was also consistent with reports from Wong *et al.* who demonstrated that combined cardiac and lung POCUS screening modified anesthetic plans in two-thirds of surgical patients [9]. In our study, anesthesia plan changes were observed in nearly one-quarter of patients, most frequently involving escalation to invasive hemodynamic monitoring, modification of anesthesia technique, or targeted cardiology consultation. Comparable rates of plan modification have been documented in large perioperative rescue echocardiography series, such as Markin *et al.* where management changed in 59% of cases [7] and in intraoperative FoCUS studies, where new findings prompted hemodynamic adjustments in unstable patients [8]. Patients with modified anesthesia plans experienced fewer intraoperative hypotensive episodes and reduced unplanned ICU admissions. Similar benefits were noted in a multicenter randomized trial in emergency non-cardiac surgery, where preoperative ultrasound-guided optimization was associated with improved perioperative stability and reduced short-term mortality [1]. Conversely, the PREOP-FOCUS randomized trial in elective high-risk surgery did not demonstrate significant improvements in composite outcomes [2], possibly reflecting differences in patient selection, the timing of POCUS application and integration of findings into care pathways.

Our study specifically targeted a high-risk population defined by Revised Cardiac Risk Index (RCRI) score and documented cardiac comorbidities, a strategy supported by prior research showing higher diagnostic yield when FoCUS is applied selectively rather than universally [5, 6 and 11]. The use of a standardized protocol for image acquisition and interpretation, performed by anesthesiologists with formal POCUS training, likely contributed to the diagnostic accuracy and clinical impact observed. Similar emphasis on structured scanning and interpretation is highlighted in consensus frameworks for perioperative POCUS [12, 13] and in algorithmic hemodynamic assessment approaches [14, 15]. Beyond its diagnostic capabilities, POCUS serves as a decision-making adjunct in perioperative cardiovascular optimization. Reviews have outlined its role in guiding fluid therapy, vasopressor initiation and postoperative monitoring [10, 16 and 17] in aligning preoperative findings with American Heart Association (AHA) and American College of Cardiology (ACC) guideline-based cardiovascular risk management [18, 19]. Furthermore, its value extends to complex perioperative contexts, including the COVID-19 era, where rapid, bedside imaging minimized patient transfers and reduced infection risk [20]. Despite these promising findings, limitations exist. The observational design cannot establish causality between POCUS-driven management changes and improved outcomes. Our sample size, while sufficient to detect differences in intraoperative stability and ICU admissions, may have been underpowered for rare but serious postoperative cardiac events. Follow-up was limited to 30 days, restricting insights into longer-term cardiovascular outcomes. Future multicenter randomized controlled trials should focus on high-risk surgical patients, incorporate standardized POCUS protocols and define clinically meaningful outcome measures. Such trials could clarify whether the management benefits observed in this and prior observational studies translate into measurable reductions in perioperative morbidity and mortality. In summary, our findings support the integration of preoperative cardiac POCUS into high-risk non-cardiac surgical pathways as a rapid, non-invasive tool that can reveal occult pathology, guide anesthesia planning and potentially improve perioperative hemodynamic stability.

## Conclusion:

Preoperative cardiac point-of-care ultrasound (POCUS) identified significant, previously unrecognized pathology in a substantial proportion of high-risk non-cardiac surgery patients. Incorporating POCUS findings into anesthetic planning led to targeted management changes associated with improved perioperative hemodynamic stability. Thus, we show adopting preoperative cardiac POCUS as a routine adjunct in the evaluation of high-risk surgical patients.
